# Targeting SARS-CoV-2 Non-Structural Proteins

**DOI:** 10.3390/ijms241613002

**Published:** 2023-08-20

**Authors:** Donald Tam, Ana C. Lorenzo-Leal, Luis Ricardo Hernández, Horacio Bach

**Affiliations:** 1 Division of Infectious Disease, Department of Medicine, University of British Columbia, 2660 Oak Street, Vancouver, BC V6H 3Z6, Canada; donaldt1@student.ubc.ca (D.T.); anacecylole@gmail.com (A.C.L.-L.); 2 Laboratorio de Investigación Fitoquímica, Departamento de Ciencias Químico Biológicas, Universidad de las Américas Puebla, Ex Hacienda Sta. Catarina Mártir S/N, San Andrés Cholula 72810, Mexico; luisr.hernandez@udlap.mx

**Keywords:** SARS-CoV-2, non-structural protein, virulence factors, drug targeting, multi-drug regimen, replication transcription complex, RNA capping machinery

## Abstract

Severe acute respiratory syndrome coronavirus 2 (SARS-CoV-2) is an enveloped respiratory β coronavirus that causes coronavirus disease (COVID-19), leading to a deadly pandemic that has claimed millions of lives worldwide. Like other coronaviruses, the SARS-CoV-2 genome also codes for non-structural proteins (NSPs). These NSPs are found within open reading frame 1a (ORF1a) and open reading frame 1ab (ORF1ab) of the SARS-CoV-2 genome and encode NSP1 to NSP11 and NSP12 to NSP16, respectively. This study aimed to collect the available literature regarding NSP inhibitors. In addition, we searched the natural product database looking for similar structures. The results showed that similar structures could be tested as potential inhibitors of the NSPs.

## 1. Introduction

Severe acute respiratory syndrome coronavirus 2 (SARS-CoV-2) is an enveloped respiratory β coronavirus that causes coronavirus disease (COVID-19), leading to a deadly pandemic that has claimed millions of lives worldwide [1].

The genome of SARS-CoV-2 is an approximately 29.9 kbp long positive-sense single-stranded RNA molecule with a 5′-cap and 3′-poly-A tail that encodes structural proteins, non-structural proteins (NSP), and accessory proteins [2,3].

The spherical SARS-CoV-2 is decorated by the Spike (S) protein, a critical protein that facilitates the entry of the virus into the host cell [4]. The spike protein must bind to the angiotensin-converting enzyme 2 (ACE2) receptor and undergoes cleavage by the transmembrane serine protease 2 (TMPRSS2) [2]. Upon successfully entering a susceptible host cell expressing the ACE2 receptor and TMPRSS2, the virus can begin translating its genome almost immediately without any additional modifications due to its mature positive-sense RNA genome [2].

Consistent with other coronaviruses such as SARS-CoV-1, SARS-CoV-2 has structural proteins, including the S protein, the membrane protein (M), the envelope protein (E), and the nucleocapsid protein (N) [2]. As mentioned above, the viral S protein binds to the ACE2 receptor on the surface of host epithelial cells to gain access to the cytoplasm [5]. The M protein is involved in viral morphogenesis and budding. In addition, it stabilizes the interaction between the nucleocapsid and RNA for proper virion assembly [5]. Lastly, the E protein serves primarily as an additional barrier but can also act as a viroporin ion channel to release virions from the virally infected cell into the surrounding environment [2,5].

Within the SARS-CoV-2 virion, N proteins form a ribonucleoprotein (RNP) complex with the viral genome to package the RNA into a helical ribonucleoprotein structure [2,5].

**Figure 1:**
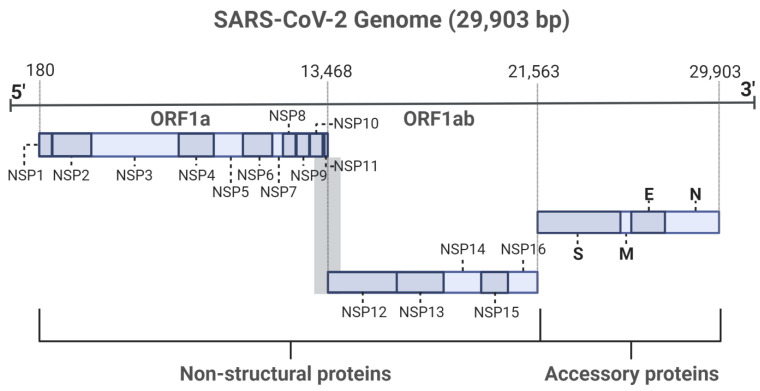
SARS-CoV-2 genome, non-structural proteins, and accessory proteins. S, spike protein; M, membrane protein; E, envelope protein; N, nucleocapsid protein. Created with BioRender.com and adapted from [6].

Like other coronaviruses, the SARS-CoV-2 genome also codes for non-structural proteins (NSPs) [2]. These NSPs are found within open reading frame 1a (ORF1a) and open reading frame 1b (ORF1ab) of the SARS-CoV-2 genome and encode NSP1 to NSP11 and NSP12 to NSP16 (Figure 1), respectively [2,3]. The open reading frame of the viral genome can be translated independently in the case of ORF1a to produce the polyprotein 1a (pp1a, NSP1 to NSP11), or it can include both open reading frames in succession to produce polyprotein 1ab (pp1ab, NSP12 to NSP16) by the host ribosome (Figure 1) [2,3]. Something unique about the SARS-CoV-2 genome is that the reading frame for NSP12 to NSP16 differs from NSP1 to NSP11. To adjust for this difference, the virus uses part of NSP11 (13 bp), which belongs to ORF1a, to shift the reading frame. As such, part of the NSP12 sequence overlaps with the NSP11 sequence, resulting in the final protein being called pp1ab despite the protein sequence being composed of ~99% pp1b and ~1% pp1a.

Following the translation of pp1a and pp1ab, these polyproteins are cleaved by two different virally encoded proteases, the papain-like protease (PLpro), also known as NSP3 and the 3-chymotrypsin-like protease (3CLpro) otherwise known as NSP5, to produce the 16 NSPs that play different roles in propagating the virus [2]. These roles include viral replication, genomic processing, and disruption of cellular processes to evade the host’s innate immune system.

To efficiently propagate the virus within the host cell, certain NSPs, such as NSP7, NSP8, and NSP12, can work in unison to form a replication transcription complex (RTC) that can create copies of the viral genome using the positive sense RNA template packaged in the virion [5]. This complex is further supported by other proteins, including a primer for RNA synthesis (NSP9), a helicase to unwind the RNA (NSP13), and an exonuclease to correct mismatched bases (NSP14) [5,7,8,9,10].

To ensure that viral replication proceeds uninterrupted, NSP6 can form a heterodimer with NSP3 (NSP3-NSP6) or NSP4 (NSP4-NSP6) to compartmentalize this process by forming double-membrane vesicles (DMV) and autophagosomes [11,12]. Once the RNA products are synthesized, they undergo a series of post-transcriptional modifications mediated by the viral proteins NSP10, NSP12, NSP13, NSP14, and NSP16, adding a 5′ mRNA cap to mimic host mRNAs. At the same time, the genome itself encodes the poly-A tail [13,14,15].

Although viral replication is necessary for viral propagation, there also needs to be other factors encoded in the viral genome that can protect viral replication by interfering with the innate immune system. One common strategy the virus employs is dampening the innate immune response. It can achieve this by interrupting autophagy and interferon pathways to disrupt the synthesis of intracellular mediators [5,16,17,18,19,20]. The virus can also impact host replication by blocking ribosome entry sites, cleaving host mRNAs, and preventing cargo transport into and out of the nucleus [21,22].

Altogether, these virulence factors encoded by NSPs play a major role in disrupting host replication while promoting the synthesis and assembly of new viral products. They are also essential for disrupting the innate immune response to prevent the host from mounting an attack against the virus, which can limit the infection. As such, identifying how these NSPs work to repurpose drugs to target viral replication and the viral inhibition of the innate immune system to allow the host to mount a more robust immune response is paramount. Similarly, it is also important to develop a multi-drug regimen that can achieve the above purpose while limiting the probability of inadvertently promoting the selection of drug-resistant strains of the virus through selective pressures.

This study aimed to collect the available literature regarding NSP inhibitors. In addition, we searched the natural product database looking for similar structures. The results showed that similar structures could be tested as potential inhibitors of the NSPs.

## 2. Description of the NSPs Present in the SARS-CoV-2 Genome

As mentioned above, the genome of SARS-CoV-2 contains 16 NSPs (Table 1). Below is a brief description of the proteins and their function to understand how they can be targeted.

### 2.1. NSP1

NSP1 inhibits the translation of host proteins by binding to the mRNA binding pocket of the 40S ribosome via its C-terminal domain [36,37]. This prevents the synthesis of important proteins involved in the innate immune response, including IFN-b, IFN-g1, IL-8, and retinoic acid-inducible gene 1, which trigger apoptosis [21,38]. The N-terminal region of NSP1 upregulates the translation of viral mRNAs by binding to the 5′ UTR stem-loop 1 region [21,39]. NSP1 recruits exonucleases that cleave host mRNA and uncapped mRNA transcripts at their internal ribosome entry sites [40]. NSP1 has also been shown to block host mRNA transcripts from exiting the nucleus by binding to the NXF1-NXT1 heterodimer docking complex involved in the nuclear export of host mRNAs through the nuclear pore complex [41]. Altogether, this virulence factor incapacitates the ability of the host cell to mount an innate immune response while enhancing viral replication [21].

### 2.2. NSP2

Attempts to determine the function of NSP2 in the host were performed by in situ biotinylation [24]. However, although results showed that NSP2 interacted with the host proteins prohibitin 1 (PHB1) and prohibitin 2 (PHB2), a validation of the results using immunoblotting of the immunoprecipitated proteins with an anti-NSP2 antibody (not available) could not be proven. PHB 1 and 2 are involved in different cell activities, such as the morphology of mitochondria [42] and transcription factor regulation [43]. Interestingly, PHB1 has been reported as a receptor facilitating the entry of Chikungunya and Dengue 2 viruses [44,45].

### 2.3. NSP3

This papain-like protease (PLpro) protein cleaves the N-terminal region of pp1a to release NSP1 to NSP3 from the polyprotein [14]. It is also involved in the cleavage of proteinaceous post-translational modifications on host proteins involved in the innate antiviral responses, further dampening the immune response Field [14]. PLpro can also regulate the innate immune response by cleaving ubiquitin-like interferon-stimulated gene 15 (*ISG15*) from interferon response factor 3 (IRF3) to inhibit the IFN I pathway [14,18,19].

### 2.4. NSP4

NSP4 is a transmembrane protein that forms part of the viral replication complex within the virally infected cell [25]. It is involved in modifying the ER membranes to form DMV in conjunction with NSP6 to compartmentalize viral replication and viral assembly, acting as an additional barrier against intracellular antiviral responses [25].

### 2.5. NSP5

NSP5 is a 3-cysteine-like protease (3Clpro), the main viral protease of SARS-CoV-2 [26]. It cleaves at 11 specific sites after glutamine residues along pp1a and pp1ab to release NSP4 to NSP16 in their intermediate or mature forms [26,46].

### 2.6. NSP6

NSP6 is a transmembrane protein that interacts with NSP3 and NSP4 to induce the formation of endoplasmic reticulum (ER)-derived autophagosomes and DMVs [47]. These autophagosomes and DMVs serve as a site for replication transcription complexes (RTCs) to form, which are necessary for viral replication [25,47]. It is also involved in modulating ER stress by binding to the host sigma receptor that is involved in limiting the production of autophagosomes and autolysosomes to disrupt viral replication [48]. NSP6 has also been implicated in activating host autophagy pathways through the omegasome pathway to promote the assembly of viral replicase proteins and the degradation of immunomodulatory proteins [47]. NSP6 also directly inhibits the cleavage-mediated activation of vacuolar proton pump components such as ATP6AP1 to impair lysosomal acidification and trigger inflammasome-mediated pyroptosis [49].

### 2.7. NSP7

NSP7 is an accessory factor protein that forms a heterotetramer with NSP8 and NSP12 to assemble the RTC needed for RNA synthesis [50].

### 2.8. NSP8

NSP8 is another accessory factor protein that forms a homodimer with itself and forms a part of the larger RTC complex alongside NSP7 and NSP12, which is necessary for RNA synthesis [50]. It has also been shown to play a role in suppressing IFN-a signaling pathways to further progress disease pathogenesis [51].

### 2.9. NSP9

NSP9 is a single-stranded RNA-binding dimeric replicase protein that binds to and activates NSP8 as a cofactor [7]. NSP9 also plays a significant role in RNA synthesis by acting as a primer for NSP12 after being modified by the NiRAN domain of NSP12 by transferring a nucleotide monophosphate to the N-terminus of NSP9 [7,9]. In the host cell, NSP9 localizes around the ER membrane, which associates with the nucleoporin 62 (NUP62), a structural protein that forms part of the nuclear pore complex, to prevent the import of p65 into the nucleus [22]. This mislocalization of p65 leads to decreased NF-kb-regulated gene expression, resulting in a dampened immune response [22].

### 2.10. NSP10

NSP10 is a growth-factor-like protein with two zinc-binding motifs that allow it to act as a cofactor for NSP16 to aid in capping viral mRNA transcripts [52]. This capping of viral mRNA transcripts increases the rate of replication by the RTC and allows the transcript to evade the host RNases that cleave uncapped mRNA transcripts [29]. NSP10 also acts as a costimulatory factor to NSP14 to enhance its exonuclease activity to remove mismatched bases, and it also allosterically activates NSP14 to aid in mRNA capping [29,53].

### 2.11. NSP11

NSP11 is a short protein that shares identical homology to the first segment of NSP12 [30]. It is an intrinsically disordered protein that sits near the junction of pp1a and pp1b. It may be involved in host cytosolic membrane affinity interactions [30]. It is also suggested that NSP11 may play a role in ribosomal frameshifting to adjust the reading frame by −1 as the reading frame for the NSPs in pp1a and pp1ab are different [30]. NSP11 is also shown to regulate endoribonuclease activity and is necessary for viral replication [30].

### 2.12. NSP12

NSP12 is an RNA-dependent RNA polymerase (RdRP) protein that acts as the core of the RTC, which binds one NSP7 and two NSP8 molecules to stabilize the RNA binding region [54], allowing viral replication to proceed [55]. The primary RNA polymerase produces nascent ssRNA from the viral RNA template [56]. NSP12 also possesses an N-terminal nidovirus RdRP-associated nucleotidyltransferase (NiRAN) domain that transfers a GMP moiety to the 5′ pp-RNA to form a 5′ Gppp-RNA cap that NSP14 can methylate in the second step of mRNA capping [29].

### 2.13. NSP13

NSP13 is an ATP-dependent RdRP containing an ATP-binding helicase core domain and a zinc-binding domain involved in unwinding the RNA during replication and the transcription of complex RNAs with secondary and tertiary structures [57,58]. It also acts as an RTPase that removes the 5′ gamma phosphate from the mRNA to produce a 5′ pp-RNA in the first step of mRNA capping [59].

### 2.14. NSP14

NSP14 is a Zn-dependent exoribonuclease that proofreads and removes mismatched nucleotides in a 3′-5′ direction incorrectly added by the RNA polymerase during genome replication [29,53,60]. This exoribonuclease domain is stabilized by the zinc-finger domain of NSP10, which increases its ability to excise nucleotides [61]. NSP14 also possesses an N7-guanine methyltransferase domain that adds a methyl group to the N7 position of the 5′ Gppp-RNA intermediate in the presence of an S-adenosylmethionine methyl donor during the second step of mRNA capping [13].

### 2.15. NSP15

NSP15 is an Mn^2+^-dependent nidoviral RNA uridylate-specific endoribonuclease (NendoU) with 3 domains: an N-terminal oligomerization domain, a central domain, and a C-terminal catalytic domain [62,63]. The catalytic domain cleaves the nucleotides of single-stranded and double-stranded RNA molecules at the 3′ end of uridylates to produce 2′-3′ phosphodiester and 5′-hydroxyl termini [62,63]. NendoUs are highly conserved in coronaviruses, and they avoid immune detection by cleaving the poly-U sequences on their negative-sense viral RNA templates to avoid detection by MDA5 pattern recognition receptors [62,64]. They also act against the innate immune system by suppressing IFN-IFN-α/β-associated pathways that lead to the formation of certain cytoplasmic stress granules [65].

### 2.16. NSP16

NSP16 is a 2′-O-ribose methyltransferase that requires an S-adenosylmethionine (SAM) methyl donor that promotes the association of NSP16 to its cofactor, NSP10 [13,14,15]. This increases the affinity of the NSP16-NSP10 complex to mRNA transcripts and allows the complex to add a methyl group to the 2′-O-ribose of the 5′ cap in the final step of mRNA capping [66]. This allows the viral mRNA to evade being detected by host MDA5 and RIG-I receptors that recognize and degrade unprocessed mRNA transcripts.

## 3. Targeting NSPs with Small Molecule Inhibitors

### 3.1. NSP1

NSP1 has two functional domains that can be targeted with drugs: the C-terminal and the N-terminal domains. Genomic sequence analyses of SARS-CoV-2 have shown that less than 3% of NSP1 sequences displayed a mutation, indicating that it is a highly conserved protein with remarkable conservation within the C-terminal domain [21]. This highly conserved region of NSP1 usually plays a role in suppressing the host immune response. It has also been shown that patients infected with variants of SARS-CoV-2 that had deletions in the helix–loop–helix region of the C-terminal domain of NSP1 had lower viral loads, corresponding to less clinically severe outcomes [38].

Some drugs that can target the C-terminal domain include montelukast sodium hydrate and mitoxantrone dihydrochloride. The N-terminal domain can also be targeted indirectly. For example, the N-terminal domain of NSP1 normally interacts with the stem-loop 1 (SL1) binding region on the 5′ UTR of the mRNA transcript to increase its translation rate [39]. Thus, by targeting this highly conserved region in the 5′ UTR, the binding of the NSP1 to the SL1 region can be prevented to decrease the viral translation of its mRNA. Some drugs have been shown to bind to the SL1 region, including glycyrrhizic acid (**1**), lobaric acid (**2**), garcinolic acid (**3**), and tirilazad (**4**).

Montelukast (**5**) is a leukotriene receptor antagonist shown to lower cytokine release and lung inflammation, reducing clinical deterioration in patients admitted with COVID-19 [63]. This reduction in infection severity may be due to montelukast binding to the C-terminal end of NSP1 to alleviate the inhibition of host protein synthesis, resulting in an increased immune response that lowers viral replication, as seen in the decreased expression of viral spike protein in virally infected HEK293T-ACE2 and Vero E6 cells [5,21]. As an FDA-approved drug for treating asthma, montelukast could be a candidate for drug repurposing to treat COVID-19.

Mitoxantrone dihydrochloride (MTX) (**6**) is a surface heparan sulfate inhibitor and anticancer drug with a high affinity for the C-terminal region of NSP1 [5,41,67]. MTX can also block viral entry into cells by binding to heparan sulfate and preventing the viral S protein from using it as a binding mediator [41,67].

Glycyrrhizic acid, lobaric acid, garcinolic acid, and tirilazad are a panel of inhibitors that show high affinity for the NSP1-SL1 binding region, which impedes its ability to interact with the 5′ UTR leader sequence on the viral mRNA [58,68]. This interaction between the SL1 region and the 5′ UTR is necessary and sufficient to allow the viral mRNA to avoid the virally induced translational inhibition exhibited by NSP1 and enhance its translation [39,69]. Thus, targeting this domain would make the viral mRNA a susceptible target for inhibition by its viral mechanism [39]. Although the main function of NSP1 is to impede host translation via its C-terminus, the NSP1-SL1 binding region has been shown to possibly play a role in translational regulation as well, which would make this panel of drugs or other antisense oligos that bind the 5′ UTR directly a viable target for future inhibitors [68,70].

### 3.2. NSP3

NSP3 is one of the two main proteases encoded by the SARS-CoV-2 genome. It is primarily targeted by drugs that bind competitively to either the active site to inhibit the enzyme directly or to an allosteric site to non-competitively block entry into the enzymatic pocket. Drugs such as the competitive inhibitor VIR251, previously used to treat SARS-CoV-1, have been repurposed due to the structural similarity in enzymatic sites between these two viruses [70]. The binding of VIR251 to the S4 binding pocket of PLpro prevents the cleavage of pp1a and the release of certain NSPs. On the other hand, allosteric inhibitors such as GRL0617 target specific naphthalene binding sites on PLpro that can prevent the entry of ISG15, an innate immune system regulator, from being cleaved and neutralized by PLpro [14,18,19]. This stops the protease from disrupting the innate immune response and allows the host to better clear the virus.

VIR251 (**7**) is a covalent inhibitor that targets the active PLpro (NSP3) site by binding to catalytic cysteine residues [70]. Due to SARS-CoV-1 and SARS-CoV-2 sharing a nearly identical binding pocket, this drug has been a primary candidate for drug repurposing to treat COVID-19 [71]. It occupies the S4 pocket of PLpro and forms an irreversible covalent thioether linkage with cysteine-111 [71,72]. The binding of VIR251 has been shown to inhibit PLpro completely from interacting with molecular probes, indicating that this drug could potentially block the protease from cleaving pp1a [71].

GRL-0617 is a naphthalene-based molecule that binds non-covalently to Tyr-268 on an adjacent naphthalene inhibitor binding site of PLpro to block entry of ISG15 into the active site [14,18,19]. It also increases the phosphorylation of IFN pathway intermediates such as IRF3, TBK1 and p65 in the NF-kB pathway [2,5,14,19]. GRL-0617 can also reverse the ubiquitination and ISGylation functions of PLpro to restore the IFN-I response needed to activate the innate immune system [19].

### 3.3. NSP5

NSP5 is the second protease encoded by the SARS-CoV-2 genome, and it is generally neutralized by targeting the cysteine residues in the active site with competitive inhibitors. A second class of peptide mimetic inhibitors is also used, which function by forming enzyme-inhibitor complexes with the protease to neutralize it.

Vinyl sulfone 2CN115 (**9**) is a small molecule competitive inhibitor of NSP5 that targets the catalytic residues within the active site of the cysteine protease to restore RIG-1 expression, which is needed for viral detection [5].

Another inhibitor is calpain inhibitor I (**10**), a synthetic tripeptide aldehyde that inhibits the main protease by forming a reversible tetrahedral complex with the cysteine residue of the enzyme [46,73].

In addition, alpha-ketoamide 13b (**11**) is an irreversible broad-spectrum peptide mimetic inhibitor of NSP5 [70,74]. It functions in a two-step process whereby the drug forms an enzyme–inhibitor complex. Then, it follows up with a secondary nucleophilic attack on the catalytic cysteine residue in the active site with its a-ketoamide group to form a covalent bond [74].

### 3.4. NSP6

The drugs that target the transmembrane protein NSP6 do not act on the protein itself but try to restore the pathways the viral protein affects. The autophagy pathway is one of the pathways that is hijacked by SARS-CoV-2 to assemble the DMVs needed for viral replication. Thus, by restoring the autophagic flux, which is the rate of autophagic degradation in the cell, the hope is to limit the formation of DMVs and thus reduce viral replication in the infected cell.

Metformin (**12**) is a biguanide compound that inhibits complex I of the electron transport chain to suppress ATP synthesis and activate AMP kinase (AMPK) through its phosphorylation [17]. The phosphorylation of AMPK has been shown to lower the total amount of viral mRNA, viral protein, and virions in Calu-3 and Caco-2 cells [17]. This may be due to AMPK activating autophagic pathways that decrease cell and tissue damage to lower inflammation [17,20]. This regulation could also restore autophagic flux to prevent the activation of NLRP3 inflammasomes that produce pro-inflammatory cytokines such as IL-1b and IL-18, which induce cell death [49]. However, the exact mechanism by which metformin limits viral replication is unknown.

Other similar compounds to metformin, such as the active form of vitamin D (1-a,25-dihydroxy vitamin D3) (**13**) and phytochemicals like polydatin (**14**), have also been shown to modulate NSP6 activity by controlling autophagic flux as well as limiting pyroptosis and ROS production [5,49].

### 3.5. NSP9

Conivaptan (**15**) is an arginine vasopressin antagonist identified by in silico screening as a potential drug targeting NSP9 [7]. It has been shown to be efficacious in in vitro studies for other coronaviruses, and its use has been associated with increased primary and secondary gene expression of cellular immune response mediators [75].

### 3.6. NSP10

Sofalcone (**16**) is a gastrointestinal medication that is an effective inhibitor of the NSP10-NSP14 proofreading complex and can work synergistically with the FDA-approved drug remdesivir to reduce its IC_50_ by 5-fold [76]. As SARS-CoV-2 relies heavily on the NSP10-NSP14 proofreading complex to maintain the integrity of its large genome during replication, inhibition of this complex will increase the probability of lethal mutations occurring [70]. Additionally, drugs such as remdesivir that can incorporate themselves into the viral genome as a nucleotide analog are very effective when used in combination with sofalcone because the inhibition of the proofreading complex decreases the likelihood that remdesivir will be excised and thus increases its potency [77].

### 3.7. NSP13

NSP13 is a virulence factor that functions as an ATP-dependent helicase to relieve RNA tension during replication and assists in the capping of viral mRNA. The commonly identified drugs used to target NSP13 look to competitively inhibit the ATPase domain of the NSP13 from blocking the ATP binding site and hindering the ability of the enzyme to associate with nucleic acids.

Punicalagin (**17**) is an antioxidant and anti-inflammatory drug that competitively inhibits NSP13 helicase activity [78]. As a competitive inhibitor, it occupies the ATPase domain of NSP13 to prevent ATP from binding. This binding also induces a conformational change in the nucleic acid binding domain, preventing it from associating with RNA [78].

Cepharanthine (**18**) is an alkaloid anti-inflammatory drug used to treat leukopenia and has been repurposed against SARS-CoV-2 [78]. Cepharanthine targets the ATP-binding site of NSP13 and blocks mRNA capping by preventing the addition of guanidine monophosphate by RNA GTase to the 5′ end [4]. This has been shown to lower viral load and expression of inflammatory factors, including TNF-a and IL-6, in mice models [79].

### 3.8. NSP14

As replication is a process prone to errors, viruses also need mechanisms to proofread their genome to remove nucleotides that have been wrongfully incorporated. For SARS-CoV-2, NSP14 is the protein that regulates this process while also serving a secondary role in mRNA capping. This enzyme has three domains that can be targets of drugs: the active site, the Zn binding sites, and the N7 methyltransferase domain.

Ritonavir (**19**) is a protease inhibitor that binds to the exoribonuclease active site of NSP14 to prevent the 3′ end of the substrate RNA from complexing with NSP14 [5,80]. This abolishes the proofreading capabilities of NSP14 by preventing the cleavage of mismatched bases from the viral genome, leading to lethal mutagenesis and the cessation of genomic replication [5,80].

Disulfiram (**20**) and ebselen (**21**) are zinc-ejector drugs that bind to the conserved zinc motifs on NSP14 to displace the zinc ions [61,70]. This would prevent the exoribonuclease from excising mismatched bases and increase the efficacy of nucleotide drugs that incorporate into the viral RNA genome to inhibit viral replication, such as remdesivir [61].

Sinefungin (**22**) is a pan-methyltransferase inhibitor and S-adenosylmethionine (SAM) analog that competitively inhibits the N7 methyltransferase domain of NSP14 by binding to a pocket between the SAM and RNA cap binding sites [5,13,70]. This prevents the SAM cofactor from donating a methyl group to NSP14 to methylate the RNA cap [4]. The interruption of RNA capping reduces translation efficiency. It increases the probability that the innate immune system responds to the uncapped mRNA in the cytoplasm, which can help trigger an immune response to eliminate the virus [13].

### 3.9. NSP15

Nascent mRNA transcripts without a 3′ poly-A tail are prone to degradation by enzymes in the cytosol. As a result, viruses have evolved to prevent the degradation of their viral mRNA in the cytoplasm by encoding repeating adenine residues at the 3′ end of each NSP. However, since nucleic acids are complementary, the complementary RNA strand used to replicate the viral genome contains repeating uracil residues. Since the host cell has evolved mechanisms that can detect uridine repeats, the virus has co-evolved a mechanism that can cleave these uridine repeats to lower the risk of detection by host PRRs. As NSP15 is responsible for removing uridine repeats to avoid detection by the innate immune system, drugs have been repurposed to target the active site of NSP15 to prevent the enzyme from binding to the polyuridine repeats and cleaving the viral mRNA transcripts.

Epigallocatechin gallate (EGCG) (**23**) is a natural compound derived from green tea extracts that competitively inhibits NSP15 by binding to the active site and preventing the cleavage of uridylates from the growing RNA strand [62]. EGCG has been shown to completely inhibit the replication of SARS-CoV-2 with a concentration as low as 1 μg/mL [62]. This potency may be due to EGCG also adhering to other viral proteins such as PLpro (NSP3), 3CLpro (NSP5), and RdRP (NSP12), though further experiments would need to be performed to confirm this.

Tipiracil (**24**) is a uracil derivative that inhibits thymidine phosphorylases. It has been repurposed as a competitive inhibitor of NSP15 by binding to the active site to inhibit its EndoU activity [64]. However, tipiracil alone is insufficient for blocking viral replication and proliferation. Thus, it would need to be used in a combinatorial approach with other drugs mentioned in this paper to treat COVID-19 effectively [64].

## 4. Approved Treatments

The current treatment and prevention of COVID-19 involves using primarily three different vaccines approved by the Federal Drug Administration (FDA) in the United States. These include the Pfizer-BioNTech, Moderna, and Janssen vaccines [81]. However, there are some antiviral inhibitors available that have been approved for emergency use. Currently, the FDA has authorized the usage of remdesivir (**25**), nirmaltrevir (**26**), and molnupiravir (**27**) for the treatment of COVID-19 in non-hospitalized adults, and the World Health Organization (WHO) has recommended the usage of certain drugs under conditions of non-severe illness and severe COVID-19.

In mild cases, the WHO recommends the use of a cocktail of the monoclonal antibodies cassirivimab and imdevinmab [https://www.who.int/publications/i/item/WHO-2019-nCoV-therapeutics-2022.4, accessed on 3 March 2023]. For severe COVID-19 cases, it suggests the use of tocilizumab, sarilumab, or baricitinib [https://www.who.int/publications/i/item/WHO-2019-nCoV-therapeutics-2022.4, accessed on 3 March 2023]. Thus, there is a wide variety of commercially available therapeutics for treating COVID-19. However, there are still many unknowns surrounding the mechanisms of these drugs, and further research is required to elucidate their mechanisms of action.

### 4.1. Remdesivir

Remdesivir (**25**) is a phosphoramidate prodrug metabolized within the body to produce an active nucleoside analog that causes replication to cease when added to a growing RNA strand by RdRP (NSP12) [82]. After remdesivir is incorporated into the growing RNA strand, the RdRP can add three more nucleotides before forming a translocation barrier. This translocation barrier locks the 3′ nucleotide within the substrate binding site of the polymerase and prevents new nucleotides from being added [82]. This inhibition may be due to the steric clash between the C1′-cyano group in remdesivir and the serine-861 side chain of NSP12, which impacts the positioning of the RNA within the enzyme and prevents the further addition of nucleotides [83].

### 4.2. Paxlovid

Paxlovid is a combinatorial drug treatment involving the coadministration of nirmatrelvir (**26**) and ritonavir (**18**) [84]. Nirmatrelvir functions as a competitive peptidomimetic inhibitor of the 3CLpro or main protease of SARS-CoV-2 (NSP5) by forming a reversible covalent bond with the cysteine-145 residue in the active site of the protease [85].

The role of ritonavir is to target and irreversibly block the cytochrome P450 enzyme CYP3A family responsible for oxidizing nirmatrelvir [85,86]. This will increase the bioavailability and decrease the dosage of nirmatrelvir required for treatment while reducing the potential side effects associated with its use [85,86]. Together these two drugs can inhibit the main protease of SARS-CoV-2 and prevent the cleavage of pp1ab into its NSP byproducts.

### 4.3. Molnupiravir

Molnupiravir (**26**) is an isopropyl ester prodrug similar to remdesivir in that they both must be metabolized into an active nucleoside analog [87,88]. The metabolized form of molnupiravir is NHC, and this metabolite can enter cells and be phosphorylated to produce an active ribonucleoside triphosphate (NHC-TP) that can induce RNA mutagenesis [87,88]. When NHC-TP is added to the growing negative-sense RNA strand in place of cytidine triphosphate or uridine triphosphate by RdRp, it generates an error in the viral genome [87,89]. This error, however, does not trigger the termination of RNA synthesis due to the stable bond formed between NHC-TP and its respective base pair, allowing replication to continue uninterrupted [87,89,90]. Though when the negative-sense RNA template containing NHC-TP is used to create a new positive-sense RNA template, the nucleotide that is paired with NHC-TP can be either an adenosine triphosphate or a guanosine triphosphate, resulting in mutagenesis if the incorrect base was added. This positive-sense RNA template containing numerous mutations will then be unable to produce functional viral proteins, limiting the viral propagation in the host [88,90].

### 4.4. Casirivimab/Imdevinmab

Casirivimab and imdevinmab are a pair of neutralizing immunoglobulin-g1 (IgG1) human monoclonal antibodies that bind to different epitopes of the spike protein of SARS-CoV-2 [91]. These monoclonal antibodies function by blocking the receptor binding domain region to prevent the spike protein from associating with the ACE2 receptor, which inhibits viral entry into the cell by abolishing receptor-mediated endocytosis [91].

### 4.5. Tocilizumab/Sarilumab

Tocilizumab and sarilumab are a pair of MAbs that bind to membrane-bound and soluble IL-6 receptors (IL-6R) [92]. Blocking IL-6Rs prevents IL-6 from exerting its pro-inflammatory effects on the immune system, which leads to the activation of the adaptive immune system. Since IL-6 levels are upregulated in patients with COVID-19 and elevated levels of this cytokine are linked to greater mortality, administering these MAbs that can antagonize the IL-6R may aid in modulating the cytokine storms and help the body better clear the infection [92].

### 4.6. Baricitinib

Baricitinib (**27**) is an ATP-dependent Janus kinase 1/2 (JAK1/2) inhibitor aimed at reducing the systemic inflammation caused by the JAK-STAT pathway during SARS-CoV-2 infection through a competitive and reversible inhibition [93].

Since JAKs are key signal transducers in the JAK-STAT pathway, which controls the transcription of multiple cytokines, inhibiting JAKs is key to lowering cytokine production [94,95]. This leads to a reduction in many inflammatory cytokines, including IL-6, TNF-a, IL-1b, IL-10, IL-8, and CXCL3, contributing to the cytokine storms seen in severe cases of COVID-19.

## 5. Similarities between Approved Treatments and Natural Products

We compared the structures of the compounds (**1**–**27**) interacting with the different NSPs to natural compounds using the NPASS database (https://bidd.group/NPASS/search.php, accessed on 15 June 2023). For the analysis of similar structures, the similarity coefficient of Tanimoto (*≥0.9*) was selected. From all the possible structures, the most similar carbon skeleton was chosen.

The natural compounds found (Table 2) could represent alternative compounds that can increase the natural product arsenal against SARS-CoV-2. Although not all the natural compounds shown in Table 2 have been tested as potential antivirals, some showed similar activities to the original compounds reported in other viral models. The natural source of the alternative inhibitors is detailed in Table 3.

For example, psoromic acid (**29**) is a β-orcinol depsidone that has been reported to inhibit Herpes Simplex virus type 1 and type 2 [96]. This report highlighted that the mechanism of inhibition is related to the viral DNA polymerase. When psoromic acid was combined with the antiviral acyclovir, superior antiviral activity was measured. Acyclovir also targets viral DNA replication by binding its converted form acyclo-GTP to the DNA polymerase [97].

Regarding 1-a,24R,25-trihydroxylcholecalciferol, it is the terminal product in 1α,25-dihydroxycholecalciferol (vitamin D, calcitriol) metabolism. It is also known as calcitroic acid and is produced upon hydroxylation of calcitriol by the CYP24A1 24-hydroxylase. This modification turns calcitriol into a more soluble molecule, facilitating its excretion in the bile and urine [98].

Although calcitroic acid has not been tested as an antiviral, its intermediate, calcitriol, has been reported to induce autophagy in human macrophages through a phosphatidylinositol 3-kinase-, ATG-5-, and Beclin-1-dependent mechanism that significantly inhibits the human immunodeficiency virus 1 replication in a dose-dependent manner [99].

The tannin chebulagic acid (**39**) has been reported as a broad-spectrum antiviral because it has been used to target different viral models [100]. Reports showed its ability to target human cytomegalovirus, hepatitis C virus, dengue virus, measles virus, and respiratory syncytial virus in a dose-dependent manner [99]. In addition, it can inhibit the replication of human enterovirus 71 [101], influenza A virus [102], and SARS-CoV-2 [103]. Several mechanisms of action have been proposed related to chebulagic acid’s activity. For example, an allosteric inhibition of the viral 3-chymotrypsin-like cysteine protease (3CLpro) [103], the inhibition of the channel influenza A virus [102] and neuraminidase-mediated viral release [104], and the inhibition of viral attachment in the human cytomegalovirus, hepatitis C virus, dengue virus, measles virus, and respiratory syncytial virus have been used as viral models [100].

On the other hand, the tannin punicalin (**40**) was shown to inhibit the replication of the episomal stage of the hepatitis B virus, limiting the formation of the pregenomic RNA in the infected cells [105]. Moreover, inhibition of the human immunodeficiency virus reverse transcriptase was reported, which also limits the replication of the virus in lymphocyte cells [106].

## 6. Treatment Management

Currently, the therapeutics recommended by the WHO for the treatment of COVID-19 are based on the severity of the disease. While this approach is effective for treating patients in the present, using mono-drug treatments can have long-lasting impacts on the evolution of viruses due to mutations in the viral genome driven by selection pressures. Using single therapeutics to treat any viral infection poses the risk of inadvertently selecting drug-resistant variants, which can render our currently available therapeutics ineffective. As such, it is important to identify possible therapeutics that can effectively neutralize and stop the viral life cycle at multiple different points to reduce the likelihood of selecting these drug-resistant variants. It is also essential to examine how multiple drugs can be used synergistically. This will increase the treatment’s efficacy while minimizing the risk of resistance, which is essential for reducing this virus to treatable endemic levels, like the common flu.

When selecting a drug for treatment, the first factor is identifying how well the target site is conserved between different virus variants. Regions of the viral genome that are generally well conserved are those involved in replication, as mutations in these areas directly affect the ability of the virus to generate more copies of itself. These include the two main virally encoded proteases, the RTC and the proofreading exoribonuclease, which are prime targets as the viability of the virus would also be compromised if it acquired mutations in these regions to combat our therapeutics. Thus, by targeting these conserved sites, we can prevent the processing of the polyprotein into its functional subunits and impair viral replication by reducing the reliability of viral replication.

When selecting candidates for combinatorial drug treatments, it is also important to consider how well the drugs work in tandem and whether there are any reductions in efficacy when they are co-administered. This can be achieved through in silico screenings that can model whether there are any adverse interactions between the drugs and viable candidates and can then be later validated through in vitro and in vivo experiments.

Considering the above factors, a drug cocktail to inhibit viral protein synthesis could consist of VIR251, Paxlovid, and tirilazad. In this formulation, VIR251 would inhibit PLpro to block the cleavage of pp1a, while Paxlovid would inhibit 3CLpro to stop pp1ab from being cleaved into the NSPs, limiting viral protein activity. If both protease inhibitors fail to prevent the cleavage of the polyproteins, tirilazad could act upstream on NSP1 to inhibit the synthesis of viral mRNAs to prevent the synthesis of more viral polyproteins. Another proposed multi-drug regimen focused more on inhibiting viral replication could include sofalcone, disulfiram, and remdesivir.

Sofalcone and disulfiram are both inhibitors of the viral proofreading mechanism that target the enzyme at different target sites. Since these drugs lower the fidelity of replication, they synergize effectively with remdesivir by preventing the removal of the nucleotide analog from the growing RNA strand. This, in turn, would increase the likelihood of chain termination occurring.

Although these multi-drug regimens may be an effective form of treatment, some limitations may prevent their implementation in practice. Issues concerning the tolerance of taking multiple drugs simultaneously can be a limiting factor. Also, accounting for changes in bioavailability and determining the effective and inhibitory dosages may be challenging with the increased number of factors at play. Thus, it may be more feasible to approach treatment with single drugs; acknowledging the benefits and drawbacks of using multi-drug treatments is important to consider for improving treatments in the future.

Moreover, some similar structures found in the database (**28**–**45**) have shown antiviral activities in other viral models. In this regard, validation is required to address their inhibitory effects on SARS-CoV-2.

## Figures and Tables

**Table 1:** NSPs produced by the SARS-CoV-2 genome (accession number P0DTC1).

Protein	MW (kDa)	Amino Acid Length	Function	References
NSP1	19.8	180	Binding 40S ribosome	[23]
NSP2	70.5	638	Potential binding to prohibitins	[24]
NSP3	217.3	1945	Papain-like protease	[14]
NSP4	56.2	500	Formation of double-membrane vesicles (DMVs) replication organelles	[25]
NSP5	33.8	306	3C-like protease	[26]
NSP6	33.0	290	Promotes the formation of double-membrane vesicles in infected cells, ER/cytosolic protein	[25]
NSP7	9.2	83	Essential cofactor that binds to NSP12, forming a complex that stabilizes the polymerase domain	[27]
NSP8	21.9	198	Extends the template RNA-binding surface	[27]
NSP9	12.4	113	Involved in viral RNA synthesis	[28]
NSP10	14.8	139	Viral replication	[29]
NSP11	1.3	13	Unknown	[30]
NSP12	106.7	932	Viral replication	[31]
NSP13	66.9	601	Helicase	[32]
NSP14	59.8	527	3′ to 5′ ExoN activity	[33]
NSP15	38.8	346	RNA processing	[34]
NSP16	33.3	298	mRNA capping	[35]

**Table 2:** Similar structures of natural compounds compared to reported inhibitors of NSPs (**1–27**). structures to NSP inhibitors.

Compound No.	Structure	Similar No.	Similar Structures
**1** Glycyrrhizic acid CID: 14982. CHEMBL441687. CAS: 1405-86-3. SMILE:C[C@@]12CC[C@@H]3[C@](C)(CC[C@H](O[C@H]4O[C@H](C(=O)O)[C@@H](O)[C@H](O)[C@H]4O[C@@H]4O[C@H](C(=O)O)[C@@H](O)[C@H](O)[C@H]4O)C3(C)C)[C@H]1C(=O)C=C1[C@@H]3C[C@](C)(CC[C@]3(C)CC[C@]12C)C(=O)O	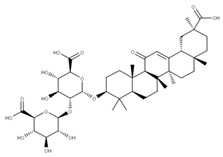	**28** 28-O-β-D-Glucopyranosyloleanolic acid 3-O-β-D-galactopyranosyl (1->3)-β-D-glucuronopyranoside-6-O-n-butyl ester. NPC104372. CID: 16099401. CHEMBL493640. SMILE: CCCCOC(=O)[C@@H]1[C@H]([C@@H]([C@H]([C@H](O[C@H]2CC[C@@]3(C)[C@@H](CC[C@]4(C)[C@@H]3CC=C3[C@@H]5CC(C)(C)CC[C@@]5(CC[C@@]43C)C(=O)O[C@H]3[C@@H]([C@H]([C@@H]([C@@H](CO)O3)O)O)O)C2(C)C)O1)O)O[C@H]1[C@@H]([C@H]([C@H]([C@@H](CO)O1)O)O)O)O	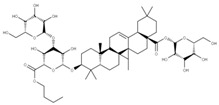
**2** Lobaric acid CID: 73157. CHEMBL551842. CAS: 522-53-2 SMILE:O=C1Oc2cc(O)c(C(=O)O)c(CCCCC)c2Oc2cc(OC)cc(c12)C(=O)CCCC	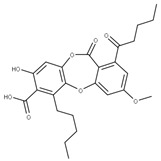	**29** Psoromic acid NPC10576. CID: 23725. CHEMBL176570. CAS: 7299-11-8 SMILE: Cc1cc(c(C=O)c2c1C(=O)Oc1c(C)c(cc(c1O2)C(=O)O)OC)[O-]	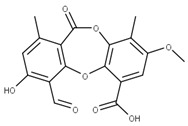
**3** Garcinolic acid CID: 6857794. CHEMBL1456870. SMILE: CC1(Oc2c(C\C=C(/C)C)c3OC45C(CCC=C4C(=O)c3c(O)c2C=C1)C(C)(C)O[C@@]5(C\C=C(/C)C(=O)O)C(=O)O)CC\C=C(/C)C	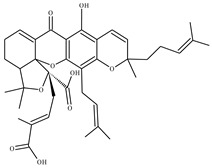	**30** Neogambogic acid CID: 125071. CAS: 93772-31-7. SMILE: CC(=CCCC1(CC(C2=C(C3=C(C(=C2O1)CC=C(C)C)OC45C6CC(C=C4C3=O)C(=O)C5(OC6(C)C)CC=C(C)C(=O)O)O)O)C)C	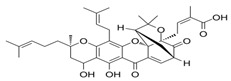
		**31** 10-α-Hydroxyepigambogic acid CID: 74429469. SMILE: CC(=CCCC1(C=CC2=C(C3=C(C(=C2O1)CC=C(C)C)OC45C6CC(C(C4C3=O)O)C(=O)C5(OC6(C)C)CC=C(C)C(=O)O)O)C)C	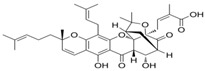
		**32** Gambogenific acid NPC162248. CID: 25208911. CHEMBL558353. SMILE: C/C(=CCc1c2O[C@H](Cc2c2c(c1O)C(=O)C1=C[C@@H]3C[C@@H]4[C@@]1(O2)[C@@](C/C=C(C(=O)O)/C)(OC4(C)C)C3=O)C(O)(C)C)/CCC=C(C)C.	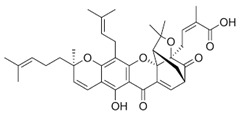
		**33** Nimbolinin C CID: 10794274. LTS0151616. SMILE: CC1=C2[C@H](C[C@H]1C3=COC=C3)O[C@H](C[C@H]4[C@]2([C@@H]([C@H]5[C@@H]6[C@@]4([C@H](C[C@H]([C@]6(CO5)C)OC(=O)C)OC(=O)/C=C/C7=CC=CC=C7)C)O)C)OC	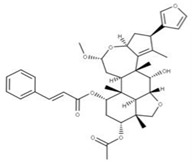
		**34** Scortechinone R NPC473607. CID: 44559271. CHEMBL447070. SMILE: CO[C@]12C=C3C(=O)c4c(O[C@@]53[C@H](C1)C(O[C@@]5(C2=O)C/C=C(C(=O)O)/C)(C)C)c1c(c(c4O)CC(C(=C)C)O)O[C@@H](C1(C)C)C	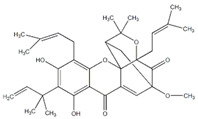
**5** Montelukast CID: 5281040. CHEMBL787. CAS: 158966-92-8. SMILE: OC(C)(C)c1ccccc1CC[C@@H](SCC1(CC(=O)O)CC1)c1cccc(c1)/C=C/c1nc2cc(Cl)ccc2cc1	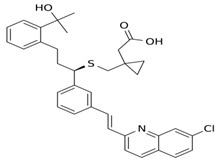	No compound found	
**6** Mitoxantrone dihydrochloride CID: 164946820. CHEMBL5078683. SMILE: O=C(N[C@@H](CN)C(=O)NCC(=O)NC/C=C/C(=O)OC)[C@H](CCc1sc2ccccc2n1)NC(=O)C	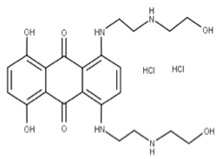	No compound found	
**7** VIR251 CID: 164946820. CHEMBL5078683. SMILE: O=C(N[C@@H](CN)C(=O)NCC(=O)NC/C=C/C(=O)OC)[C@H](CCc1sc2ccccc2n1)NC(=O)C	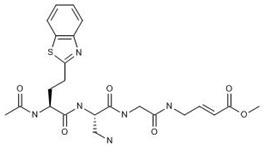	No compound found	
**8** GRL-0617 CID: 24941262. CHEMBL549695. CAS: 1093070-16-6 SMILE: O=C(N[C@H](C)c1cccc2ccccc12)c1cc(N)ccc1C	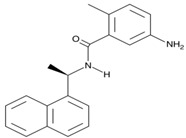	No compound found	
**9** Vinyl sulfone-2CN115 CID: 156621389. SMILE: CS(=O)(=O)\C=C\[C@H](CCC(=O)N)NC(=O)[C@@H](NC(=O)OCc1cccc(C#C)c1)CC(C)C	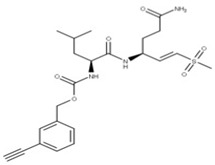	No compound found	
**10** Calpain inhibitor I CID: 443118. CHEMBL304784. CAS: 110044-82-1. SMILE: CC(=O)N[C@@H](CC(C)C)C(=O)N[C@@H](CC(C)C)C(=O)N[C@@H](CCCC)C=O	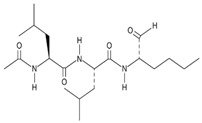	No compound found	
**11** α-ketoamide 13b CID: 146026181. CHEMBL5075209. SMILE: O=C(OC(C)(C)C)NC1=CC=CN(C1=O)[C@@H](CC1CC1)C(=O)N[C@@H](C[C@@H]1CCNC1=O)C(=O)C(=O)NCc1ccccc1	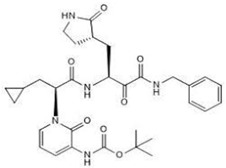	No compound found	
**12** Metformin CID: 152743144. CAS: 657-24-9. SMILE: N=C(\N=C(/N)N)N(C)C	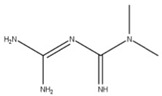	No compound found	
**13** Vitamin D3 derivative CID: 52080453. CHEMBL846. CAS: 32222-06-3. SMILE: C[C@]12CCC\C(=C/C=C3/C[C@@H](O)C[C@H](O)C3=C)[C@@H]1CC[C@@H]2[C@H](C)CCCC(C)(O)C	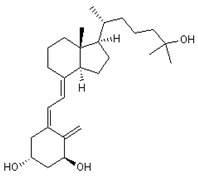	**35** 25-Hydroxycholecalciferol (calcifediol) NPC27395. CID: 5283731. CHEMBL1040. CAS: 19356-17-3 SMILE: O[C@H]1CCC(=C)/C(=CC=C2/CCC[C@]3([C@H]2CC[C@@H]3[C@@H](CCCC(O)(C)C)C)C)/C1	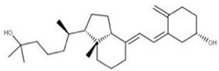
		**36** 1α,24R,25-Trihydroxylcholecalciferol NPC320548. CID: 6446280. CHEMBL3351075. CAS: 56142-94-0. SMILE: O[C@H]1C[C@H](O)C(=C)/C(=CC=C2/CCC[C@]3([C@H]2CC[C@@H]3[C@@H](CC[C@H](C(O)(C)C)O)C)C)/C1	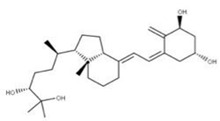
**14** Conivaptan CID: 151171. CHEMBL1755. CAS: 210101-16-9. SMILE: O=C(N1CCc2[NH]c(C)nc2c2ccccc12)c1ccc(NC(=O)c2ccccc2c2ccccc2)cc1	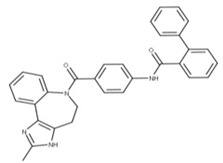	No compound found	
**15** Sofalcone CID: 5282219. CHEMBL1441961. CAS: 64506-49-6. SMILE: C/C(C)=C/COc1cc(OCC(=O)O)c(cc1)C(=O)/C=C/c1ccc(cc1)OC\C=C(/C)C	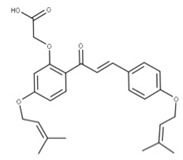	**37** 4-Geranyloxy-2,6 dihydroxybenzophenone NPC124269. CID: 44575287. CHEMBL510517. SMILE: C/C(=CCOc1cc(O)c(c(c1)O)C(=O)c1ccccc1)/CCC=C(C)C	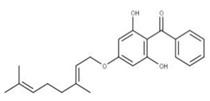
		**38** Cordoin NPC240593. CID: 5961410. CHEMBL450771. CAS: 38965-74-1 SMILE: CC(=CCOc1ccc(C(=O)/C=C/c2ccccc2)c(c1)O)C	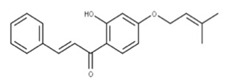
**16** Punicalagin CID: 44584733. CAS: 65995-63-3. SMILE: OC1O[C@@H]2COC(=O)c3cc(O)c(O)c(O)c3c3c(O)c(O)c4OC(=O)c5c6c(OC(=O)c3c46)c(O)c(O)c5c3c(cc(O)c(O)c3O)C(=O)O[C@H]2[C@@H]2OC(=O)c3cc(O)c(O)c(O)c3c3c(cc(O)c(O)c3O)C(=O)O[C@@H]12	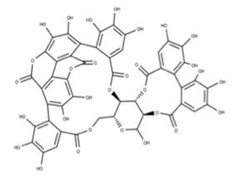	**39** Chebulagic acid NPC119094. CID: 442674. CHEMBL525240 SMILE: OC(=O)C[C@@H]1C(=O)O[C@@H]2[C@H]3COC(=O)c4cc(O)c(c(c4c4c(C(=O)O[C@@H]2[C@@H](OC(=O)c2c5[C@H]1[C@H](O)C(=O)Oc5c(c(c2)O)O)[C@@H](O3)OC(=O)c1cc(O)c(c(c1)O)O)cc(O)c(c4O)O)O)O	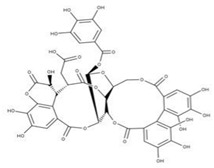
		**40** Punicalin NPC123259. CID: 5464368. CHEMBL502440. CAS: 65995-64-4. SMILE: O[C@@H]1O[C@@H]2COC(=O)c3cc(O)c(c(c3c3c(O)c(O)c4c5c3c(=O)oc3c(c(c(c6c(C(=O)O[C@H]2[C@@H]([C@H]1O)O)cc(O)c(c6O)O)c(c(=O)o4)c53)O)O)O)O	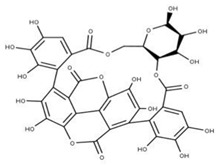
**17** Cepharanthine CID: 10206. CHEMBL449782. CAS: 481-49-2 SMILE: COc1cc2CCN(C)[C@@H]3Cc4ccc(OC)c(Oc5ccc(cc5)C[C@@H]5N(C)CCc6cc7OCOc7c(Oc1cc32)c56)c4	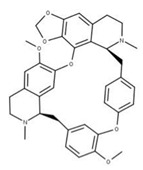	**41** Isochondodendrine NPC104196. CID: 197726 CHEMBL1169628. CAS: 477-62-3. SMILE: CN1CCc2cc(c(c3c2[C@H]1Cc1ccc(cc1)Oc1c2c(CCN(C)[C@@H]2Cc2ccc(cc2)O3)cc(c1O)OC)O)OC	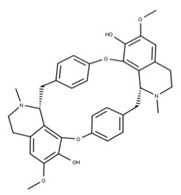
		**42** Isotetrandrine NPC10908. CID: 5351212. CHEMBL504757. CAS: 26137-48-4. SMILE: CN1CCc2cc(c3cc2[C@H]1Cc1ccc(cc1)Oc1cc(ccc1OC)C[C@H]1c2c(CCN1C)cc(c(c2O3)OC)OC)OC	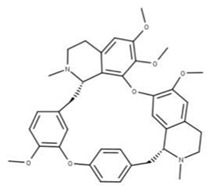
**18** Ritonavir CID: 392622. CHEMBL163. CAS: 155213-67-5. SMILE: CN(Cc1nc(sc1)C(C)C)C(=O)N[C@H](C(=O)N[C@H](C[C@H](O)[C@@H](NC(=O)OCc1scnc1)Cc1ccccc1)Cc1ccccc1)C(C)C	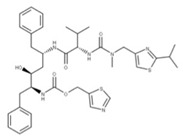	No compound found	
**19** Disulfiram CID: 3117. CHEMBL964. CAS: 97-77-8. SMILE: CCN(CC)C(=S)SSC(=S)N(CC)CC	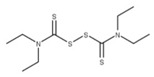	No compound found	
**20** Ebselen CID: 3194. CHEMBL51085. CAS: 60940-34-3. SMILE: O=C1N([Se]c2ccccc12)c1ccccc1	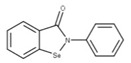	No compound found	
**21** Sinefungin CID: 65482. CHEMBL1214186. CAS: 58944-73-3. SMILE: O=C(O)[C@@H](N)CC[C@H](N)C[C@H]1O[C@@H](n2cnc3c2ncnc3N)[C@H](O)[C@@H]1O	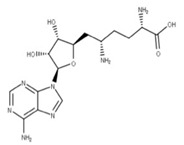	**43** S-Adenosylhomocysteine NPC324484. CID: 439155. CHEMBL418052. CAS: 979-92-0. SMILE: C1=NC(=C2C(=N1)N(C=N2)C3C(C(C(O3)CSCCC(C(=O)[O-])[NH3+])O)O)N	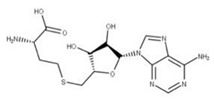
**22** Epigallocatechin gallate CID: 65064. CHEMBL297453. CAS: 989-51-5. SMILE: O=C(O[C@@H]1Cc2c(cc(O)cc2O)O[C@@H]1c1cc(O)c(O)c(O)c1)c1cc(O)c(O)c(O)c1	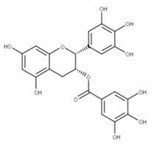	**44** 3,4,5-Trihydroxy-benzoic acid (2R,3S)-5,7-dihydroxy-2-(3,4,5-trihydroxy-phenyl)-chroman-3-Yl ester NPC104983. CID: 5276890. CHEMBL126079. CAS: 5127-64-0. SMILE: Oc1cc(O)c2c(c1)O[C@@H]([C@H](C2)OC(=O)c1cc(O)c(c(c1)O)O)c1cc(O)c(c(c1)O)O	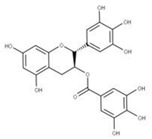
		**45** (-)-Epigallocatechin gallate NPC291948. CID: 2824823. CHEMBL338988 SMILE: Oc1cc(O)c2c(c1)O[C@H]([C@H](C2)OC(=O)c1cc(O)c(c(c1)O)O)c1cc(O)c(c(c1)O)O	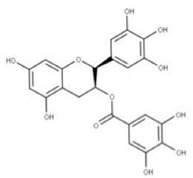
**23** Tipiracil CID: 6323266. CHEMBL235668. CAS: 183204-74-2. SMILE: O=C1NC(=O)C(Cl)=C(CN2CCCC2=N)N1	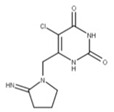	No compound found	
**24** Remdesivir CID: 121304016. CHEMBL4065616. CAS: 1809249-37-3. SMILE: O=[P@](Oc1ccccc1)(OC[C@H]1O[C@](C#N)([C@H](O)[C@@H]1O)c1ccc2n1ncnc2N)N[C@@H](C)C(=O)OCC(CC)CC	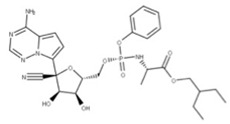	No compound found	
**25** Nirmatrelvir CID: 155903259. CHEMBL4802135. CAS: 2628280-40-8. SMILE: CC1(C)[C@@H]2[C@H](N(C[C@@H]21)C(=O)[C@@H](NC(=O)C(F)(F)F)C(C)(C)C)C(=O)N[C@H](C#N)C[C@@H]1CCNC1=O	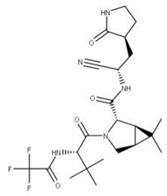	No compound found	
**26** Molnupiravir CID: 145996610. CHEMBL4650320. CAS: 2492423-29-5. SMILE: ONC=1C=CN(C(=O)N=1)[C@@H]1O[C@H](COC(=O)C(C)C)[C@@H](O)[C@H]1O	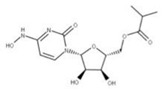	No compound found	
**27** Baricitinib CID: 44205240. CHEMBL2105759. CAS: 1187594-09-7. SMILE: O=S(=O)(CC)N1CC(CC#N)(C1)n1cc(cn1)c1ncnc2[NH]ccc21	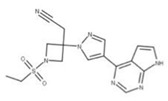	No compound found	

Database compound IDs: CID: PubChem; CHEMBL: European Molecular Biology Laboratory; CAS: Chemical Abstracts Service; LTS: Lotus; NPC: Natural Product Activity & Species Source Database (NPASS).

**Table 3:** Sources of natural compounds with similar structures to NSP inhibitors.

Compound	Similar Structures	Source
**28**	28-O-b-D-Glucopyranosyloleanolic acid 3-O-b-D-galactopyranosyl(1->3)-b-D-glucuronopyranoside-6-O-n-butyl ester	*Calendula officinalis* (P)
	Licorice saponins (several compounds)	*Glycyrrhiza uralensis* (P)
**29**	Psoromic acid	*Adiantum capillus-veneris* (P) *Aesculus pavia* (P) *Antrodia sinuosa* (F) *Calea nelsonii* (P) *Colletotrichum acutatum* (F) *Cephalocroton cordofanus* (P) *Dendrobium moniliforme* (P) *Lamium galeobdolon* subsp. *montanum* (P)
**30**	Neogambogic acid	*Garcinia hanburyi* (P)
**31**	10-a-Hydroxyepigambogic acid	*Garcinia hanburyi* (P)
**32**	Gambogenific acid	*Garcinia hanburyi* (P)
**33**	Nimbolinin C	*Melia azedarach* (P)
**34**	Scortechinone R	*Garcinia scortechinii* (P)
**35**	25-Hydroxycholecalciferol (calcifediol)	*Homo sapiens* (M)
**36**	1-a,24R,25-Trihydroxylcholecalciferol	*Homo sapiens* (M)
**37**	4-Geranyloxy-2,6-dihydroxybenzophenone	*Cratoxylum sumatranum* (P) *Pseudosuberites hyalinus* (S) *Ribes rubrum* (P) *Scorzonera judaica* (P) *Tovomita longifolia* (P) *Verbena brasiliensis* (P)
**38**	Cordoin	*Crotalaria dura* (P) *Duguetia staudtii* (P) *Fasciospongia cavernosa* (S) *Liquidambar orientalis* (P) *Millettia erythrocalyx* (P) *Salvia phlomoides* (P) *Solidago arguta* (P)
**39**	Chebulagic acid	*Bupleurum chinense* (P) *Excoecaria agallocha* (P) *Leonurus sibiricus* (P) *Lumbricus terrestris* (W) *Phyllanthus emblica* (P)
**40**	Punicalin	*Aspergillus candidus* (F) *Beauveria felina* (F) *Conyza canadensis* (P) *Cirsium japonicum* (P) *Cladiella krempfi* (C) *Garcinia hombroniana* (P) *Punica granatum* (P)
**41**	Isochondodendrine	*Coffea canephora* (P) *Collybia albuminosa* (F) *Cyclea barbata* (P) *Cyclea racemose* (P) *Isolona ghesquiereina* (P) *Megaselia halterata* (F) *Mustela putorius furo* (M)
**42**	Isotetrandrine	*Cornus officinalis* (P) *Cyclea barbata* (P) *Melianthus major* (P) *Pestalotiopsis disseminate* (F) *Stephania cepharantha* (P) *Stephania tetrandra* (P) *Thalictrum faberi* (P)
**43**	S-Adenosylhomocysteine	*Homo sapiens* (M)
**44**	3,4,5-Trihydroxy-benzoic acid (2R,3S)-5,7-dihydroxy-2-(3,4,5-trihydroxy-phenyl)-chroman-3-Yl ester	*Corylus avellana* (P) *Morus alba* (P) *Paeonia lactiflora* (P)
**45**	(-)-Epigallocatechin gallate	*Camellia sinensis* (P)

C, coral; F, fungus; M, mammal; P, plant; S, sponge; W, worm.

## Data Availability

Requests for data will be considered by the corresponding author.

## References

[1] Bchetnia M., Girard C., Duchaine C., Laprise C. (2020). The outbreak of the novel severe acute respiratory syndrome coronavirus 2 (SARS-CoV-2): A review of the current global status. J. Infect. Public Health.

[2] Brant A.C., Tian W., Majerciak V., Yang W., Zheng Z.-M. (2021). SARS-CoV-2: From its discovery to genome structure, transcription, and replication. Cell Biosci..

[3] Yadav R., Chaudhary J.K., Jain N., Chaudhary P.K., Khanra S., Dhamija P., Sharma A., Kumar A., Handu S. (2021). Role of structural and non-structural proteins and therapeutic targets of SARS-CoV-2 for COVID-19. Cells.

[4] Yan R., Zhang Y., Li Y., Ye F., Guo Y., Xia L., Zhong X., Chi X., Zhou Q. (2021). Structural basis for the different states of the spike protein of SARS-CoV-2 in complex with ACE2. Cell Res..

[5] Low Z.Y., Zabidi N.Z., Yip A.J.W., Puniyamurti A., Chow V.T.K., Lal S.K. (2022). SARS-CoV-2 non-structural proteins and their roles in host immune evasion. Viruses.

[6] Redondo N., Zaldívar-López S., Garrido J.J., Montoya M. (2021). SARS-CoV-2 Accessory Proteins in Viral Pathogenesis: Knowns and Unknowns. Front Immunol..

[7] Chandel V., Sharma P.P., Raj S., Choudhari R., Rathi B., Kumar D. (2022). Structure-based drug repurposing for targeting Nsp9 replicase and spike proteins of severe acute respiratory syndrome coronavirus 2. J. Biomol. Struct. Dyn..

[8] Raj R. (2021). Analysis of non-structural proteins, NSPs of SARS-CoV-2 as targets for computational drug designing. Biochem. Biophys. Rep..

[9] Wang B., Svetlov D., Artsimovitch I. (2021). NMPylation and de-NMPylation of SARS-CoV-2 nsp9 by the NiRAN domain. Nucleic Acids Res..

[10] Yapasert R., Khaw-On P., Banjerdpongchai R. (2021). Coronavirus infection-associated cell death signaling and potential therapeutic targets. Molecules.

[11] Wolff G., Melia C.E., Snijder E.J., Bárcena M. (2020). Double-Membrane Vesicles as Platforms for Viral Replication. Trends Microbiol..

[12] Klein S., Cortese M., Winter S.L., Wachsmuth-Melm M., Neufeldt C.J., Cerikan B., Stanifer M.L., Boulant S., Bartenschlager R., Chlanda P. (2020). SARS-CoV-2 structure and replication characterized by in situ cryo-electron tomography. Nat. Commun..

[13] Ahmed-Belkacem R., Sutto-Ortiz P., Guiraud M., Canard B., Vasseur J.-J., Decroly E., Debart F. (2020). Synthesis of adenine dinucleosides SAM analogs as specific inhibitors of SARS-CoV nsp14 RNA cap guanine-N7-methyltransferase. Eur. J. Med. Chem..

[14] Shin D., Mukherjee R., Grewe D., Bojkova D., Baek K., Bhattacharya A., Schulz L., Widera M., Mehdipour A.R., Tascher G. (2020). Papain-like protease regulates SARS-CoV-2 viral spread and innate immunity. Nature.

[15] Tazikeh-Lemeski E., Moradi S., Raoufi R., Shahlaei M., Janlou M.A.M., Zolghadri S. (2021). Targeting SARS-CoV-2 non-structural protein 16: A virtual drug repurposing study. J. Biomol. Struct. Dyn..

[16] Santerre M., Arjona S.P., Allen C.N., Shcherbik N., Sawaya B.E. (2021). Why do SARS-CoV-2 NSPs rush to the ER?. J. Neurol..

[17] Parthasarathy H., Tandel D., Siddiqui A.H., Harshan K.H. (2022). Metformin suppresses SARS-CoV-2 in cell culture. Virus Res..

[18] Pitsillou E., Liang J., Ververis K., Lim K.W., Hung A., Karagiannis T.C. (2020). Identification of small molecule inhibitors of the deubiquitinating activity of the SARS-CoV-2 papain-like protease: In silico molecular docking studies and in vitro enzymatic activity assay. Front. Chem..

[19] Rao P., Patel R., Shukla A., Parmar P., Rawal R.M., Saraf M., Goswami D. (2022). Identifying structural-functional analogue of GRL0617, the only well-established inhibitor for papain-like protease (PLpro) of SARS-CoV2 from the pool of fungal metabolites using docking and molecular dynamics simulation. Mol. Divers..

[20] Xian H., Liu Y., Rundberg Nilsson A., Gatchalian R., Crother T.R., Tourtellotte W.G., Zhang Y., Aleman-Muench G.R., Lewis G., Chen W. (2021). Metformin inhibition of mitochondrial ATP and DNA synthesis abrogates NLRP3 inflammasome activation and pulmonary inflammation. Immunity.

[21] Afsar M., Narayan R., Akhtar M.N., Das D., Rahil H., Nagaraj S.K., Eswarappa S.M., Tripathi S., Hussain T. (2022). Drug targeting Nsp1-ribosomal complex shows antiviral activity against SARS-CoV-2. eLife.

[22] Makiyama K., Hazawa M., Kobayashi A., Lim K., Voon D.C., Wong R.W. (2022). NSP9 of SARS-CoV-2 attenuates nuclear transport by hampering nucleoporin 62 dynamics and functions in host cells. Biochem. Biophys. Res Commun..

[23] Lapointe C.P., Grosely R., Johnson A.G., Wang J., Fernández I.S., Puglisi J.D. (2021). Dynamic competition between SARS-CoV-2 NSP1 and mRNA on the human ribosome inhibits translation initiation. Proc. Natl. Acad. Sci. USA.

[24] Cornillez-Ty C.T., Liao L., Yates J.R., Kuhn P., Buchmeier M.J. (2009). Severe Acute Respiratory Syndrome Coronavirus Nonstructural Protein 2 Interacts with a Host Protein Complex Involved in Mitochondrial Biogenesis and Intracellular Signaling. J. Virol..

[25] Angelini M.M., Akhlaghpour M., Neuman B.W., Buchmeier M.J. (2013). Severe Acute Respiratory Syndrome Coronavirus Nonstructural Proteins 3, 4, and 6 Induce Double-Membrane Vesicles. mBio.

[26] Roe M.K., Junod N.A., Young A.R., Beachboard D.C., Stobart C.C. (2021). Targeting novel structural and functional features of coronavirus protease nsp5 (3CLpro, Mpro) in the age of COVID-19. J. Gen. Virol..

[27] Kirchdoerfer R.N., Ward A.B. (2019). Structure of the SARS-CoV nsp12 polymerase bound to nsp7 and nsp8 co-factors. Nat. Commun..

[28] Sutton G., Fry E., Carter L., Sainsbury S., Walter T., Nettleship J., Berrow N., Owens R., Gilbert R., Davidson A. (2004). The nsp9 Replicase Protein of SARS-Coronavirus, Structure and Functional Insights. Structure.

[29] Bouvet M., Imbert I., Subissi L., Gluais L., Canard B., Decroly E. (2012). RNA 3′-end mismatch excision by the severe acute respiratory syndrome coronavirus nonstructural protein nsp10/nsp14 exoribonuclease complex. Proc. Natl. Acad. Sci. USA.

[30] Gadhave K., Kumar P., Kumar A., Bhardwaj T., Garg N., Giri R. (2021). Conformational dynamics of 13 amino acids long NSP11 of SARS-CoV-2 under membrane mimetics and different solvent conditions. Microb. Pathog..

[31] Suryawanshi R.K., Koganti R., Agelidis A., Patil C.D., Shukla D. (2021). Dysregulation of Cell Signaling by SARS-CoV-2. Trends Microbiol..

[32] Newman J.A., Douangamath A., Yadzani S., Yosaatmadja Y., Aimon A., Brandão-Neto J., Dunnett L., Gorrie-stone T., Skyner R., Fearon D. (2021). Structure, mechanism and crystallographic fragment screening of the SARS-CoV-2 NSP13 helicase. Nat. Commun..

[33] Hsu J.C.-C., Laurent-Rolle M., Pawlak J.B., Wilen C.B., Cresswell P. (2021). Translational shutdown and evasion of the innate immune response by SARS-CoV-2 NSP14 protein. Proc. Natl. Acad. Sci. USA.

[34] Kim Y., Jedrzejczak R., Maltseva N.I., Wilamowski M., Endres M., Godzik A., Michalska K., Joachimiak A. (2020). Crystal structure of Nsp15 endoribonuclease NendoU from SARS-CoV-2. Protein Sci..

[35] Sk M.F., Jonniya N.A., Roy R., Poddar S., Kar P. (2020). Computational Investigation of Structural Dynamics of SARS-CoV-2 Methyltransferase-Stimulatory Factor Heterodimer nsp16/nsp10 Bound to the Cofactor SAM. Front. Mol. Biosci..

[36] Thoms M., Buschauer R., Ameismeier M., Koepke L., Denk T., Hirschenberger M., Kratzat H., Hayn M., Mackens-Kiani T., Cheng J. (2020). Structural basis for translational shutdown and immune evasion by the Nsp1 protein of SARS-CoV-2. Science.

[37] Schubert K., Karousis E.D., Jomaa A., Scaiola A., Echeverria B., Gurzeler L.-A., Leibundgut M., Thiel V., Mühlemann O., Ban N. (2020). SARS-CoV-2 Nsp1 binds the ribosomal mRNA channel to inhibit translation. Nat. Struct. Mol. Biol..

[38] Kao H.-T., Orry A., Palfreyman M.G., Porton B. (2022). Synergistic interactions of repurposed drugs that inhibit Nsp1, a major virulence factor for COVID-19. Sci. Rep..

[39] Vora S.M., Fontana P., Mao T., Leger V., Zhang Y., Fu T.-M., Lieberman J., Gehrke L., Shi M., Wang L. (2022). Targeting stem-loop 1 of the SARS-CoV-2 5′ UTR to suppress viral translation and Nsp1 evasion. Proc. Natl. Acad. Sci USA.

[40] Gaglia M.M., Covarrubias S., Wong W., Glaunsinger B.A. (2012). A Common Strategy for Host RNA Degradation by Divergent Viruses. J. Virol..

[41] Zhang K., Miorin L., Makio T., Dehghan I., Gao S., Xie Y., Zhong H., Esparza M., Kehrer T., Kumar A. (2021). Nsp1 protein of SARS-CoV-2 disrupts the mRNA export machinery to inhibit host gene expression. Sci. Adv..

[42] Tatsuta T., Model K., Langer T. (2005). Formation of membrane-bound ring complexes by prohibitins in mitochondria. Mol. Biol. Cell..

[43] Gamble S.C., Chotai D., Odontiadis M., Dart D.A., Brooke G.N., Powell S.M., Reebye V., Varela-Carver A., Kawano Y., Waxman J. (2007). Prohibitin, a protein downregulated by androgens, represses androgen receptor activity. Oncogene.

[44] Wintachai P., Wikan N., Kuadkitkan A., Jaimipuk T., Ubol S., Pulmanausahakul R., Auewarakul P., Kasinrerk W., Weng W.-Y., Panyasrivanit M. (2012). Identification of prohibitin as a Chikungunya virus receptor protein. J. Med. Virol..

[45] Kuadkitkan A., Wikan N., Fongsaran C., Smith D.R. (2010). Identification and characterization of prohibitin as a receptor protein mediating DENV-2 entry into insect cells. Virology.

[46] Milligan J.C., Zeisner T.U., Papageorgiou G., Joshi D., Soudy C., Ulferts R., Wu M., Lim C.T., Tan K.W., Weissmann F. (2021). Identifying SARS-CoV-2 antiviral compounds by screening for small molecule inhibitors of Nsp5 main protease. Biochem. J..

[47] Cottam E.M., Maier H.J., Manifava M., Vaux L.C., Chandra-Schoenfelder P., Gerner W., Britton P., Ktistakis N.T., Wileman T. (2011). Coronavirus nsp6 proteins generate autophagosomes from the endoplasmic reticulum via an omegasome intermediate. Autophagy.

[48] Cottam E.M., Whelband M.C., Wileman T. (2014). Coronavirus NSP6 restricts autophagosome expansion. Autophagy.

[49] Sun X., Liu Y., Huang Z., Xu W., Hu W., Yi L., Liu Z., Chan H., Zeng J., Liu X. (2022). SARS-CoV-2 non-structural protein 6 triggers NLRP3-dependent pyroptosis by targeting ATP6AP1. Cell Death Differ..

[50] Te Velthuis A.J.W., van den Worm S.H.E., Snijder E.J. (2012). The SARS-coronavirus nsp7+nsp8 complex is a unique multimeric RNA polymerase capable of both de novo initiation and primer extension. Nucleic Acids Res..

[51] Xia H., Cao Z., Xie X., Zhang X., Chen J.Y.-C., Wang H., Menachery V.D., Rajsbaum R., Shi P.-Y. (2020). Evasion of Type I Interferon by SARS-CoV-2. Cell Rep..

[52] Decroly E., Debarnot C., Ferron F., Bouvet M., Coutard B., Imbert I., Gluais L., Papageorgiou N., Sharff A., Bricogne G. (2011). Crystal Structure and Functional Analysis of the SARS-Coronavirus RNA Cap 2′-O-Methyltransferase nsp10/nsp16 Complex. PLoS Pathog..

[53] Ma Y., Wu L., Shaw N., Gao Y., Wang J., Sun Y., Lou Z., Yan L., Zhang R., Rao Z. (2015). Structural basis and functional analysis of the SARS coronavirus nsp14–nsp10 complex. Proc. Natl. Acad. Sci. USA.

[54] Peng Q., Peng R., Yuan B., Zhao J., Wang M., Wang X., Wang Q., Sun Y., Fan Z., Qi J. (2020). Structural and Biochemical Characterization of the nsp12-nsp7-nsp8 Core Polymerase Complex from SARS-CoV-2. Cell Rep..

[55] Hillen H.S., Kokic G., Farnung L., Dienemann C., Tegunov D., Cramer P. (2020). Structure of replicating SARS-CoV-2 polymerase. Nature.

[56] Subissi L., Posthuma C.C., Collet A., Zevenhoven-Dobbe J.C., Gorbalenya A.E., Decroly E., Snijder E.J., Canard B., Imbert I. (2014). One severe acute respiratory syndrome coronavirus protein complex integrates processive RNA polymerase and exonuclease activities. Proc. Natl. Acad. Sci. USA.

[57] Chen J., Malone B., Llewellyn E., Grasso M., Shelton P.M.M., Olinares P.D.B., Maruthi K., Eng E.T., Vatandaslar H., Chait B.T. (2020). Structural Basis for Helicase-Polymerase Coupling in the SARS-CoV-2 Replication-Transcription Complex. Cell.

[58] Yan L., Zhang Y., Ge J., Zheng L., Gao Y., Wang T., Jia Z., Wang H., Huang Y., Li M. (2020). Architecture of a SARS-CoV-2 mini replication and transcription complex. Nat. Commun..

[59] Ivanov K.A., Ziebuhr J. (2004). Human Coronavirus 229E Nonstructural Protein 13: Characterization of Duplex-Unwinding, Nucleoside Triphosphatase, and RNA 5′-Triphosphatase Activities. J. Virol..

[60] Chen Y., Cai H., Pan J., Xiang N., Tien P., Ahola T., Guo D. (2009). Functional screen reveals SARS coronavirus nonstructural protein nsp14 as a novel cap N7 methyltransferase. Proc. Natl. Acad. Sci. USA.

[61] Chen T., Fei C.-Y., Chen Y.-P., Sargsyan K., Chang C.-P., Yuan H.S., Lim C. (2021). Synergistic Inhibition of SARS-CoV-2 Replication Using Disulfiram/Ebselen and Remdesivir. ACS Pharmacol. Transl. Sci..

[62] Hong S., Seo S.H., Woo S.-J., Kwon Y., Song M., Ha N.-C. (2021). Epigallocatechin gallate inhibits the uridylate-specific endoribonuclease Nsp15 and efficiently neutralizes the SARS-CoV-2 strain. J. Agric. Food Chem..

[63] Khan A.R., Misdary C., Yegya-Raman N., Kim S., Narayanan N., Siddiqui S., Salgame P., Radbel J., Groote F.D., Michel C. (2022). Montelukast in hospitalized patients diagnosed with COVID-19. J. Asthma.

[64] Kim Y., Wower J., Maltseva N., Chang C., Jedrzejczak R., Wilamowski M., Kang S., Nicolaescu V., Randall G., Michalska K. (2021). Tipiracil binds to uridine site and inhibits Nsp15 endoribonuclease NendoU from SARS-CoV-2. Commun. Biol..

[65] Nakagawa K., Narayanan K., Wada M., Makino S. (2018). Inhibition of Stress Granule Formation by Middle East Respiratory Syndrome Coronavirus 4a Accessory Protein Facilitates Viral Translation, Leading to Efficient Virus Replication. J. Virol..

[66] Zeng C., Wu A., Wang Y., Xu S., Tang Y., Jin X., Wang S., Qin L., Sun Y., Fan C. (2016). Identification and Characterization of a Ribose 2′-O-Methyltransferase Encoded by the Ronivirus Branch of Nidovirales. J. Virol..

[67] Kumar P., Bhardwaj T., Giri R. (2022). Mitoxantrone dihydrochloride, an FDA approved drug, binds with SARS-CoV-2 NSP1 C-terminal. RSC Adv..

[68] Vankadari N., Jeyasankar N.N., Lopes W.J. (2020). Structure of the SARS-CoV-2 Nsp1/5′-untranslated region complex and implications for potential therapeutic targets, a vaccine, and virulence. J. Phys. Chem. Lett..

[69] Bujanic L., Shevchuk O., von Kügelgen N., Kalinina A., Ludwik K., Koppstein D., Zerna N., Sickmann A., Chekulaeva M. (2022). The key features of SARS-CoV-2 leader and NSP1 required for viral escape of NSP1-mediated repression. RNA.

[70] Yan W., Zheng Y., Zeng X., He B., Cheng W. (2022). Structural biology of SARS-CoV-2: Open the door for novel therapies. Signal Transduct. Target Ther..

[71] Rut W., Lv Z., Zmudzinski M., Patchett S., Nayak D., Snipas S.J., El Oualid F., Huang T.T., Bekes M., Drag M. (2020). Activity profiling and crystal structures of inhibitor-bound SARS-CoV-2 papain-like protease: A framework for anti-COVID-19 drug design. Sci. Adv..

[72] Hognon C., Marazzi M., García-Iriepa C. (2022). Atomistic-level description of the covalent inhibition of SARS-CoV-2 papain-like protease. Int. J. Mol. Sci..

[73] Banerjee S. (2021). An insight into the interaction between α-ketoamide- based inhibitor and coronavirus main protease: A detailed in silico study. Biophys. Chem..

[74] Frecer V., Miertus S. (2020). Antiviral agents against COVID-19: Structure-based design of specific peptidomimetic inhibitors of SARS-CoV-2 main protease. RSC Adv..

[75] Khater S., Kumar P., Dasgupta N., Das G., Ray S., Prakash A. (2021). Combining SARS-CoV-2 proofreading exonuclease and RNA-dependent RNA polymerase inhibitors as a strategy to combat COVID-19: A high-throughput in silico screening. Front. Microbiol..

[76] Lin L., Wang Y., Li Q., Hu M., Shi Y. (2022). Novel SARS-CoV-2 therapeutic targets: RNA proofreading complex and virus-induced senescence. Cell Death Differ..

[77] Rona G., Zeke A., Miwatani-Minter B., de Vries M., Kaur R., Schinlever A., Garcia S.F., Goldberg H.V., Wang H., Hinds T.R. (2022). The NSP14/NSP10 RNA repair complex as a Pan-coronavirus therapeutic target. Cell Death Differ..

[78] Lu L., Peng Y., Yao H., Wang Y., Li J., Yang Y., Lin Z. (2022). Punicalagin as an allosteric NSP13 helicase inhibitor potently suppresses SARS-CoV-2 replication in vitro. Antivir. Res..

[79] Fan H., He S.-T., Han P., Hong B., Liu K., Li M., Wang S., Tong Y. (2022). Cepharanthine: A promising old drug against SARS-CoV-2. Adv. Biol..

[80] Narayanan N., Nair D.T. (2021). Ritonavir may inhibit exoribonuclease activity of nsp14 from the SARS-CoV-2 virus and potentiate the activity of chain terminating drugs. Int. J. Biol. Macromol..

[81] Mouffak S., Shubbar Q., Saleh E., El-Awady R. (2021). Recent advances in management of COVID-19: A review. Biomed. Pharmacother. Biomed. Pharmacother..

[82] Kokic G., Hillen H.S., Tegunov D., Dienemann C., Seitz F., Schmitzova J., Farnung L., Siewert A., Höbartner C., Cramer P. (2021). Mechanism of SARS-CoV-2 polymerase stalling by remdesivir. Nat. Commun..

[83] Jiang Y., Yin W., Xu H.E. (2021). RNA-dependent RNA polymerase: Structure, mechanism, and drug discovery for COVID-19. Biochem. Biophys. Res. Commun..

[84] Marzi M., Vakil M.K., Bahmanyar M., Zarenezhad E. (2022). Paxlovid: Mechanism of action, synthesis, and in silico study. BioMed. Res. Int..

[85] Joyce R.P., Hu V.W., Wang J. (2022). The history, mechanism, and perspectives of nirmatrelvir (PF-07321332): An orally bioavailable main protease inhibitor used in combination with ritonavir to reduce COVID-19-related hospitalizations. Med. Chem. Res..

[86] Loos N.H.C., Beijnen J.H., Schinkel A.H. (2022). The mechanism-based inactivation of CYP3A4 by ritonavir: What mechanism?. Int. J. Mol. Sci..

[87] Kabinger F., Stiller C., Schmitzová J., Dienemann C., Kokic G., Hillen H.S., Höbartner C., Cramer P. (2021). Mechanism of molnupiravir-induced SARS-CoV-2 mutagenesis. Nat. Struct. Mol. Biol..

[88] Saravolatz L.D., Depcinski S., Sharma M. (2023). Molnupiravir and nirmatrelvir-ritonavir: Oral coronavirus disease 2019 antiviral drugs. Clin. Infect. Dis..

[89] Gordon C.J., Tchesnokov E.P., Schinazi R.F., Götte M. (2021). Molnupiravir promotes SARS-CoV-2 mutagenesis via the RNA template. J. Biol. Chem..

[90] Tian L., Pang Z., Li M., Lou F., An X., Zhu S., Song L., Tong Y., Fan H., Fan J. (2022). Molnupiravir and its antiviral activity against COVID-19. Front. Immunol..

[91] Deeks E.D. (2021). Casirivimab/Imdevimab: First Approval. Drugs.

[92] Kim J.S., Lee J.Y., Yang J.W., Lee K.H., Effenberger M., Szpirt W., Kronbichler A., Shin J.I. (2021). Immunopathogenesis and treatment of cytokine storm in COVID-19. Theranostics.

[93] Marconi V.C., Ramanan A.V., de Bono S., Kartman C.E., Krishnan V., Liao R., Piruzeli M.L.B., Goldman J.D., Alatorre-Alexander J., de Cassia Pellegrini R. (2021). Efficacy and safety of baricitinib for the treatment of hospitalised adults with COVID-19 (COV-BARRIER): A randomised, double-blind, parallel-group, placebo-controlled phase 3 trial. Lancet Respir. Med..

[94] Hoang T.N., Pino M., Boddapati A.K., Viox E.G., Starke C.E., Upadhyay A.A., Gumber S., Nekorchuk M., Busman-Sahay K., Strongin Z. (2021). Baricitinib treatment resolves lower-airway macrophage inflammation and neutrophil recruitment in SARS-CoV-2-infected rhesus macaques. Cell.

[95] Zhang X., Zhang Y., Qiao W., Zhang J., Qi Z. (2020). Baricitinib, a drug with potential effect to prevent SARS-CoV-2 from entering target cells and control cytokine storm induced by COVID-19. Int. Immunopharmacol..

[96] Hassan S.T.S., Šudomová M., Berchová-Bímová K., Šmejkal K., Echeverría J. (2019). Psoromic Acid, a Lichen-Derived Molecule, Inhibits the Replication of HSV-1 and HSV-2, and Inactivates HSV-1 DNA Polymerase: Shedding Light on Antiherpetic Properties. Molecules.

[97] Elion G.B. (1982). Mechanism of action and selectivity of acyclovir. Am. J. Med..

[98] Jones G., Prosser D.E., Kaufmann M. (2014). Cytochrome P450-mediated metabolism of vitamin D. J. Lipid Res..

[99] Campbell G.R., Spector S.A. (2011). Hormonally Active Vitamin D3 (1α,25-Dihydroxycholecalciferol) Triggers Autophagy in Human Macrophages That Inhibits HIV-1 Infection *. J. Biol. Chem..

[100] Lin L.-T., Chen T.-Y., Lin S.-C., Chung C.-Y., Lin T.-C., Wang G.-H., Anderson R., Lin C.-C., Richardson C.D. (2013). Broad-spectrum antiviral activity of chebulagic acid and punicalagin against viruses that use glycosaminoglycans for entry. BMC Microbiol..

[101] Yang Y., Xiu J., Liu J., Zhang L., Li X., Xu Y., Qin C., Zhang L. (2013). Chebulagic Acid, a Hydrolyzable Tannin, Exhibited Antiviral Activity in Vitro and in Vivo against Human Enterovirus 71. Int. J. Mol. Sci..

[102] Duncan M.C., Onguéné P.A., Kihara I., Nebangwa D.N., Naidu M.E., Williams D.E., Balgi A.D., Andrae-Marobela K., Roberge M., Andersen R.J. (2020). Virtual Screening Identifies Chebulagic Acid as an Inhibitor of the M2(S31N) Viral Ion Channel and Influenza A Virus. Molecules.

[103] Du R., Cooper L., Chen Z., Lee H., Rong L., Cui Q. (2021). Discovery of chebulagic acid and punicalagin as novel allosteric inhibitors of SARS-CoV-2 3CLpro. Antivir. Res..

[104] Li P., Du R., Wang Y., Hou X., Wang L., Zhao X., Zhan P., Liu X., Rong L., Cui Q. (2020). Identification of Chebulinic Acid and Chebulagic Acid as Novel Influenza Viral Neuraminidase Inhibitors. Front. Microbiol..

[105] Liu C., Cai D., Zhang L., Tang W., Yan R., Guo H., Chen X. (2016). Identification of hydrolyzable tannins (punicalagin, punicalin and geraniin) as novel inhibitors of hepatitis B virus covalently closed circular DNA. Antivir. Res..

[106] Nonaka G., Nishioka I., Nishizawa M., Yamagishi T., Kashiwada Y., Dutschman G.E., Bodner A.J., Kilkuskie R.E., Cheng Y.C., Lee K.H. (1990). Anti-AIDS agents, 2: Inhibitory effects of tannins on HIV reverse transcriptase and HIV replication in H9 lymphocyte cells. J. Nat. Prod..

